# Mapping QTL for spike fertility and related traits in two doubled haploid wheat (*Triticum aestivum L*.) populations

**DOI:** 10.1186/s12870-021-03061-y

**Published:** 2021-07-26

**Authors:** Nicole Pretini, Leonardo S. Vanzetti, Ignacio I. Terrile, Guillermo Donaire, Fernanda G. González

**Affiliations:** 1 Centro de Investigaciones y Transferencia del Noroeste de la Provincia de Buenos Aires (CITNOBA, CONICET-UNNOBA-UNSADA), Monteagudo 2772 CP 2700, Pergamino, Buenos Aires, Argentina; 2 Instituto Nacional de Tecnología Agropecuaria (INTA), EEA INTA Marcos Juárez. Ruta 12 s/n CP 2850, Marcos Juárez, Córdoba, Argentina; 3 Consejo Nacional de Investigaciones Científicas y Técnicas (CONICET), Godoy Cruz 2290 CP C1425FQB, Buenos Aires, Argentina; 4 Instituto Nacional de Tecnología Agropecuaria (INTA), EEA INTA Pergamino. Ruta 32, km 4,5 CP 2700, Pergamino, Buenos Aires, Argentina

**Keywords:** Grain number, Spike length, Spikelets per spike, Fertile florets, Grain weight

## Abstract

**Background:**

In breeding programs, the selection of cultivars with the highest yield potential consisted in the selection of the yield per se, which resulted in cultivars with higher grains per spike (GN) and occasionally increased grain weight (GW) (main numerical components of the yield). In this study, quantitative trait loci (QTL) for GW, GN and spike fertility traits related to GN determination were mapped using two doubled haploid (DH) populations (Baguette Premium 11 × BioINTA 2002 and Baguette 19 × BioINTA 2002).

**Results:**

In total 305 QTL were identified for 14 traits, out of which 12 QTL were identified in more than three environments and explained more than 10% of the phenotypic variation in at least one environment. Eight hotspot regions were detected on chromosomes 1A, 2B, 3A, 5A, 5B, 7A and 7B in which at least two major and stable QTL sheared confidence intervals. QTL on two of these regions (R5A.1 and R5A.2) have previously been described, but the other six regions are novel.

**Conclusions:**

Based on the pleiotropic analysis within a robust physiological model we conclude that two hotspot genomic regions (R5A.1 and R5A.2) together with the *QGW.perg-6B* are of high relevance to be used in marker assisted selection in order to improve the spike yield potential. All the QTL identified for the spike related traits are the first step to search for their candidate genes, which will allow their better manipulation in the future.

**Supplementary Information:**

The online version contains supplementary material available at 10.1186/s12870-021-03061-y.

## Background

Wheat (*Triticum aestivum L.*) is one of the most cultivated and consumed worldwide cereals. Its production has to increase to supply the growing world population demand [1–3]. Improving the cultivar’s yield potential by breeding (i.e., the yield of a cultivar adapted to the environment, which is growing without water or nutrient deficits and with no biotic stress, [4]) is a sustainable alternative to guarantee increases in world production [5, 6]. Wheat breeding of yield potential has been based on empirical selection of yield per se due to the complexity of the character, the scarcity of knowledge and the lack of useful tools with real applicability in breeding programs [7]. This selection generally resulted in more grains per spike (GN), and hence, increased grains per unit area (no consistent trend in spikes per unit area has been reported) [8–15]. The grain weight (GW) has not shown any change with breeding; except for some recent reports in which yield potential was positively associated with its increment [16–18]. The selection process could be more efficient using molecular markers. The implementation of single nucleotide polymorphism (SNP) markers in plant breeding has increased the pace and precision of plant genetic analysis, which in turn, facilitated the implementation of crop molecular improvement [19]. SNP markers have been increasingly used for QTL mapping studies as they are the most frequent variations in the genome, and they provide a high map resolution [19, 20, 21]. Therefore, the identification, understanding and incorporation of yield related QTL could be a useful selection tool for a breeding program.

The most common approach, looking for genetic bases to further improving yield potential, is based on the numerical component analyses, GN and GW (see references quoted in Table S1). The GN is understood as the result of the total spikelets per spike (TS) and the grains per spikelet, being the former associated with the spike length (SL) and compactness of the spike (CN). Several QTL have been reported for the GW and the GN itself and their numerical components during the last years. Many studies identified stables QTL for these traits widespread in the genome (see Table S1). However, considering the IWGSC Ref. Seq. v1.0 wheat genome assembly [22], we identified QTL reported for the same trait that are located at the same position (Table 1). For example, a QTL for GW was detected in 6 studies on chromosome 7A- within 664.3–719.6 Mb [23–28] (Table 1). Two important QTL for SL were detected on chromosomes 2D and 5A [26, 29–37] (Table 1). Additionally, two QTL for TS were detected on chromosomes 5A and 7A in several studies [27, 30–32, 36, 38–41] (Table 1).

**Table 1:** Significant QTL detected in different studies that sheared interval positions according to the reference genome

Trait^a^	Chr.^b^	Position (Mb)^c^	Bibliography
**GN**	1B	16.2–55.4	Zhai et al. 2017 [42]; Li et al. 2018 [26]
	3A	705.3–749.1	Gao et al. 2015 [43]; Guan et al. 2018 [28]; Pang et al. 2020 [44]
	3B	584.6–649.5	Guo et al. 2018 [35]; Pang et al. 2020 [44]
	4A	603.3–685.0	Börner et al. 2002 [29]; Gao et al. 2015 [44]; Chen et al. 2017 [33]; Guan et al. 2018 [28]; Pang et al. 2020 [44]
	4B	16.8–98.7	Li et al. 2015 [23]; Li et al. 2018 [26]; Pang et al. 2020 [44]
	7A.1	81.1–167.3	Zhai et al. 2017 [43]; Li et al.2018 [26]
	7A.2	670.2–719.6	Li et al. 2015 [23]; Guo et al. 2017 [45]; Li et al. 2018 [26]; Guan et al. 2018 [28];
	7B	716.6–740.0	Li et al. 2018 [26]; Liu et al. 2018 [46]
**GW**	1B	566.0–679.4	Guan et al. 2018 [28]; Sukumaran et al. 2018 [47]; Gerard et al. 2019 [48]; Pang et al. 2020 [44]
	2A.1	7.5–28.3	Wang et al. 2009 [49]; Gerard et al. 2019 [48]
	2A.2	639.1–751.0	Guan et al. 2018 [28]; Ma et al. 2018 [27]; Sukumaran et al. 2018 [47]
	2D	296.4–382.2	Cuthbert et al. 2008 [50]; Wu et al. 2012 [30]; Yu et al. 2018 [51]
	3A.1	29.6–58.5	Li et al. 2018 [26]; Sukumaran et al. 2018 [47]
	3A.2	344.4–430.1	Xu et al. 2014 [31]; Wang et al. 2009 [49]
	3A.3	732.4–761.5	Cuthbert et al. 2008 [50]; Zhai et al. 2017 [42]
	4A	607.9–685.0	Ding et al. 2011 [39]; Tang et al. 2011 [52]; Gao et al. 2015 [43]; Guan et al. 2018 [28]
	4B.2	17.0–46.6	Li et al. 2018 [26]; Pang et al. 2020 [44]
	4B.1	236.7	Chen et al. 2017 [33]; Wang et al. 2011 [40]
	4D	12.8–62.5	Chen et al. 2017 [33]; Guan et al. 2018 [28]; Li et al. 2018 [26]
	5A.1	462.9–495.9	Cuthbert et al. 2008 [50]; Li et al. 2018 [26]; Sukumaran et al. 2018 [47]
	5A.2	524.2–619.0	Wang et al. 2017 [24]; Li et al. 2018 [26]; Sukumaran et al. 2018 [47]
	5A.3	656.0–682.7	Kato et al. 2000 [53]; Börner et al. 2002 [29]; Chen et al. 2017 [33]
	5B.1	27.2–47.6	Zhai et al. 2017 [43]; Pang et al. 2020 [44]
	5B.2	411.8–418.8	Zhai et al. 2016 [32]; Deng et al. 2017 [34]
	6A.1	38.4–80.0	Zhai et al. 2017 [43]; Guan et al. 2018 [28]
	6A.2	442.4–465.9	Zhai et al. 2017 [43]; Li et al. 2018 [26]
	6B	20.8–67.9	Tang et al. 2011 [52]; Li et al. 2018 [26]
	7A.1	85.7–116.0	Cuthbert et al. 2008 [50]; Tang et al. 2011 [52]
	7A.2	664.3–719.6	Li et al. 2015 [23]; Wang et al. 2017 [24]; Daba et al. 2018 [25]; Li et al. 2018 [26]; Ma et al. 2018 [27]; Guan et al. 2018 [28]
	7B	683.5–734.3	Gao et al. 2015 [43]; Li et al. 2018 [26]; Ma et al. 2018 [27]
**TS**	1A	398.6–399.5	Chen et al. 2017 [33]; Zhou et al. 2017 [54]
	2A	14.3–37.2	Ding et al. 2011 [39]; Gerard et al. 2019 [48]
	2D.1	19.6–38.3	Ma et al. 2014 [55]; Ma et al. 2018 [27]; Gerard et al. 2019 [48]
	2D.2	398.6–416.8	Zhou et al. 2017 [54]; Gerard et al. 2019 [48]
	2D.3	628.9–794.9	Zhou et al. 2017 [54]; Gerard et al. 2019 [48]
	4A	535.4–630.9	Jantasuriyarat et al. 2004 [38]; Chen et al. 2017 [33]; Ma et al. 2018 [27]
	5A	671.4–698.2	Ding et al. 2011 [39]; Wang et al. 2011 [40]; Cui et al. 2012 [41]; Wu et al. 2012 [30]
	7A	626.1–692.3	Jantasuriyarat et al. 2004 [38]; Ding et al. 2011 [39]; Xu et al. 2014 [31]; Zhai et al. 2016 [32]; Ma et al. 2018 [27]; Fan et al. 2019 [36]
	7B	622.3–718.9	Ma et al. 2018 [27]; Fan et al. 2019 [36]; Pang et al. 2020 [44]
	7D	127.3–263.0	Ma et al. 2007 [56] Yao et al. 2019 [18]
**SL**	1B	302.0–406.3	Börner et al. 2002 [29]; Jantasuriyarat et al. 2004 [38]
	2B	7.9–31.6	Cui et al. 2012 [41]; Zhai et al. 2016 [32]
	2D	17.6–99.4	Wu et al. 2012 [30]; Xu et al. 2014 [31]; Zhai et al. 2016 [32]; Chen et al. 2017 [33]; Deng et al. 2017 [34]; Li et al. 2018 [26]; Ma et al. 2019 [37]
	3B.1	25.4–52.8	Cui et al. 2012 [41]; Li et al. 2018 [26]; Pang et al. 2020 [44]
	3B.2	577.8–649.5	Guo et al. 2018 [35]; Pang et al. 2020 [44]
	4A.1	499.4–575.0	Guo et al. 2018 [35]; Li et al. 2018 [26]
	4A.2	603.3–688.1	Börner et al. 2002 [29]; Wang et al. 2011 [40]; Gao et al. 2015 [43]; Chen et al. 2017 [33]
	4B	36.7–54.7	Li et al. 2018 [26]; Pang et al. 2020 [44]
	5A	470.0–541.3	Börner et al. 2002 [29]; Zhai et al. 2016 [32]; Guo et al. 2018 [35]; Li et al. 2018 [26]; Fan et al. 2019 [36]
	6B	667.8–705.4	Deng et al. 2017 [34]; Li et al. 2018 [26]
	7D	127.3–137.3	Ma et al. 2007 [56]; Yao et al. 2019 [18]
**CN**	2D	14.4–23	Xu et al. 2014 [31]; Zhai et al. 2016 [32]
	3B	25.4–32.8	Cui et al. 2012 [41]; Pang et al. 2020 [44]
	5A	478.6–541.2	Zhai et al. 2016 [32]; Fan et al. 2019 [36]
	5B	261.8–406.9	Xu et al. 2014 [31]; Pang et al. 2020 [44]
**FS**	1A	243.4–497.5	Zhou et al. 2017 [54]; Ma et al. 2018 [27]
	1B	16.2–222.6	Zhai et al. 2016 [32]; Deng et al. 2017 [34]
	2A	16.6–56.8	Gerard et al. 2019 [48]; Ma et al. 2018 [27]
	2B	9.0–182.4	Deng et al. 2017 [34]; Ma et al. 2018[27]
	2D	19.6–88.6	Ma et al. 2018 [27]; Gerard et al. 2020 [48]
	7A	632.6–675.3	Ma et al. 2018 [27]; Fan et al. 2019 [36]

^a^*GN* grain number per spike, *GW* grain weight, *TS* total spikelets per spike, *SL* spike length, *CN* compactness of the spike, *FS* fertile spikelets per spike

^b^ Chromosome

^c^ QTL overlapped in a 50 Mb region according to the Chinese Spring RefSeq v1.0 sequence were considered as the same

From the crop physiology approach, the GN depends on the florets that reach the fertile stage at anthesis (fertile florets per spike, FF) and on the proportion of them that sets grains (grain set, GST, grains per fertile floret). Both depend on the assimilate availability, the first one for the growing spike and developing florets during the 20 days before anthesis [57–59], and the second one during the 10 days after anthesis [57, 60]. This would explain the high importance in GN and FF determination of: (i) the spike dry weight achieved at anthesis (SDW) [10, 12, 61, 62]; and (ii) the dry matter partitioned within the spike between florets/grains and spike structure parts, i.e. the fertile floret efficiency (FFE, fertile florets per g of SDW) [63] and the fruiting efficiency (FE, grains per g of SDW, or FEm grains per g of chaff at maturity) [15, 63–68]. It has been reported that in modern elite cultivars the SDW was less important to explain GN variation than the fruiting efficiency [15, 63–69]. The GST is considered to be high in relative modern cultivars (i.e., > 80% of fertile florets set grains) [10, 67, 70], but it has been recently shown that it can be as low as 60% [45, 63]. Then, the amount of assimilates partitioned within the spike to grains, to chaff (CH) or to its structures (glume, lemma, palea, awns GLPA-, and rachis R-), as well as the GST and SDW, are worthy of study. Only few reports looked for the genetic bases of these traits [26, 45, 48, 71, 72] (Table S1).

In a previous work [72], dealing with one of the DH populations used in the present study, we identified and validated the novel *QFEm.perg-3A* for FEm on chromosome 3A, and the first known QTL for FFE and FE, *QFFE.perg-5A*, located on chromosome 5A. This last QTL was also detected when the FEm was measured, agreeing with Basile et al. [71] who detected two regions within this QTL associated with FEm. Despite studying the pleiotropic effect of both *QFFE.perg-5A* and *QFEm.perg-3A* on the associated traits of spike fertility previously mentioned, we did not look for new major and stable QTL for those traits themselves.

The aim of this study is to identify stable and major QTL for the spike fertility and related traits (numerical and physiological components) and to discuss the possible pleiotropic effects among them and with the previously reported *QFEm.perg-3A* and *QFFE.perg-5A*. Two doubled haploid populations were used, Baguette Premium 11 × BioINTA 2002 (BP11xB2002) and Baguette 19 × BioINTA2002 (B19xB2002) derived from the crosses of elite cultivars adapted to the central region of the wheat-producing area of Argentina.

In the present study, we report 305 QTL for different spike fertility and related traits distributed throughout the wheat genome. Nevertheless, only 28 QTL are considered major and stable. Eight genomic regions that group some of the significant and stable QTL have been identified and their pleiotropic effects on other related traits have been also analysed. Finally, we have found two regions (R5A.1 and R5A.2) and a QTL (*QGW.perg-6B*) that resulted in a final higher spike yield.

## Results

### Genetic linkage map construction

The linkage map of BP11xB2002 consisted of 7,323 SNPs and two functional markers for the vernalization genes *Vrn-A1* [73] and *Vrn-B1* [74] (Tables S2, S3). All the SNPs represented 723 loci across the 21 wheat chromosomes. The map covered 2605.3 cM in length with an average locus spacing of 4.7 cM (Tables S2, S3). The linkage map of B19xB2002 was previously described in Pretini et al. [72]. Briefly, the B19xB2002 map consisted of 10,936 SNPs and the *Vrn-A1* and *Vrn-B1* markers. All the SNPs represented 739 loci across the 21 wheat chromosomes (Tables S4, S5). The map covered 2,221.7 cM in length with an average locus spacing of 4.3 cM (Tables S4, S5). Although the genome length of each population was similar, distribution of the markers in the three genomes was not uniform. In BP11xB2002, genomes A and B were similar with at least three times the number of polymorphic markers than in genome D, with 2,955, 3,513 and 857 markers, respectively (Table S3). In B19xB2002, the marker uneven distribution was higher with 4,126, 5,448 and 1,364 markers in genomes A, B and D, respectively (Table S5). The number of loci in genomes A and B were three times the number of loci in genome D in both populations. For BP11xB2002 there were 311 and 300 loci in genomes A and B and 113 loci in genome D whereas for B19xB2002 there were 324 and 317 loci in genomes A and B and 98 loci in genome D (Tables S3, S5).

### Phenotypic analysis

The means and ranges of the 14 studied traits across the five environments (E1 to E5) for the three parents and both DH populations are detailed in Table S6. According to BLUE values, B19 and BP11 parents had higher FF, FFTS (fertile florets per total spikelet), FFFS (fertile florets per fertile spikelet) and GN, whereas B2002 had higher SDW, SL, TS, CH, R, GLPA and GW (Table 2). BP11 showed the highest and B19 the lowest FS value, while B2002 was in-between (Table 2). All traits presented a normal distribution across each environment and BLUE values, with a transgressive segregation from both parent lines in both populations (Table 2, Table S6). The narrow-sense heritability values ranged from 0.31 to 0.86, depending on the trait and the DH population (Table 2).

**Table 2:** Means, ranges, heritability and Shapiro–Wilk test for all traits based on the BLUE values

**Trait**^**a**^	**Parental Line**			**BP11xB2002**						**B19xB2002**					
	**B2002**	**BP11**	**B19**	**Min**	**Max**	**Mean**	**SD**^**b**^	**W**^**c**^	**h**^**2**^	**Min**	**Max**	**Mean**	**SD**	**W**	**h**^**2**^
SL	98.6	93.7	87.2	86.6	122.7	101.6	7.5	0.97	0.81	79.4	105.5	94.0	6.4	0.96	0.63
TS	22.2	19.6	20.0	19.6	26.4	22.7	1.6	0.96	0.86	18.7	26.1	21.1	1.4	0.98	0.66
CN	4.8	4.5	4.4	3.8	5.4	4.5	0.3	0.96	0.78	3.1	5.6	4.5	0.4	0.97	0.63
FF	44.9	52.6	46.6	37.1	65.5	49.4	5.7	0.98	0.60	34.7	52.9	43.9	4.1	0.97	0.52
FS	18.0	18.5	16.2	16.1	22.3	19.0	1.3	0.97	0.73	14.5	19.3	17.0	1.0	0.95	0.58
FFTS	2.0	2.4	2.3	1.7	2.7	2.2	0.2	0.95	0.51	1.3	2.5	2.1	0.2	0.96	0.66
FFFS	2.4	2.8	2.8	2.2	3.1	2.6	0.2	0.95	0.58	2.0	3.0	2.5	0.2	0.96	0.68
SDW	418	359	357	305	502	408	36	0.99	0.31	279	480	370	38	0.98	0.36
R	83	67	61	58	115	79	10	0.95	0.69	52	94	72	9	0.97	0.51
GLPA	436	253	346	236	439	335	47	0.96	0.50	262	478	359	44	0.95	0.50
CH	513	315	399	307	535	414	52	0.95	0.48	323	564	431	50	0.96	0.50
GN	37.4	40.4	39.3	29.4	53.4	39.8	5.5	0.96	0.59	27.1	50.0	38.0	4.2	0.99	0.53
GW	35.8	31.1	34.0	24.2	43.3	31.8	3.8	0.97	0.72	26.6	43.5	34.6	3.8	0.95	0.39
GST	0.88	0.83	0.87	0.59	1.11	0.87	0.10	0.98	-*	0.50	1.30	0.90	0.20	0.92	0.43

^a^*SL* spike length (mm), *TS* total spikelets per spike (n° spike^−1^), *CN* compactness of the spike (mm node^−1^), *FF* fertile florets per spike (n° spike^−1^), *FS* fertile spikelets per spike (n° spike^−1^), *FFTS* fertile florets per total spikelet (n° spikelet^−1^), *FFFS* fertile florets per fertile spikelet (n° spikelet^−1^), *SDW* spike dry weight at anthesis (mg spike^−1^), *R* rachis (mg spike^−1^), GLPA: glume + lemma + palea + awns (mg spike^−1^), CH: chaff (no-grain spike dry weight at harvest, mg spike^−1^), GN: grain number per spike (n° spike^−1^), GW: grain weight (mg), GST: grain set

^b^ *SD* standard deviation

^c^ W: A modification of the test of Shapiro-Wilks for normality. Mahibbur and Govindarajulu [75]

^*^ error means square > genotype mean squares

The phenotypic performances of the GN, FF, SDW, CH and GST in both populations were already described in Pretini et al. [63]. Briefly, the range of mean values for the BP11xB2002 and B19xB2002 populations based on the BLUE values, were: (i) 29.4 to 53.4 and 27.2 to 50.0 grains per spike for GN; (ii) 37.1 to 65.5 and 34.7 to 52.9 florets per spike for FF; (iii) 305 to 502 and 279 to 480 mg per spike for SDW; (iv) 307 to 535 and 323 to 564 mg per spike for CH; and finally (v) 0.6 to 1.1 and from 0.5 to 1.3 for GST (Table 2).

In relation to the traits determining spike structure at anthesis, the highest SL, considering the agronomic environments (E1 to E3) and both populations, was observed in E1 (~ 107 mm), while the lowest SL was observed in E2 (~ 93) (Table S6). The E5 (a non-agronomic summer season) showed even a lower SL than E2 (~ 73 mm) (Table S6). The TS ranged similarly for both DH populations within the agronomic environments (from ~ 21 to 24 spikelets per spike in E2 to E1); while the lowest TS was observed in the non-agronomic environment (E5, ~ 16 spikelets per spike, Table S6). The FS ranges were also similar for both populations (from ~ 17 to 20 fertile spikelets per spike in E2 to E1) and the lowest FS was observed in the E5 (~ 11 fertile spikelets per spike, Table S6). The CN was the lowest for both populations in the E2 (4.4 mm per spikelet, Table S6); while the highest (4.6 mm) was detected in E1 for BP11xB2002 and in E5 for B19xB2002 (Table S6).

For both populations the FFTS ranged from ~ 2.0 to 2.4 fertile florets per total spikelet (E2 to E1, Table S6), while the lowest FFTS was detected in the E5 with 1.7 fertile florets per total spikelet (Table S6). Even though the range explored by the FFFS was similar for both populations, ~ 2.4 to 2.7 fertile florets per fertile spikelet, the maximum and minimum values were associated with different environments in each population (E5 to E3 in BP11xB2002 and E2 to E1 in B19xB2002, Table S6).

The CH partitioning between R and GLPA varied from 14 to 22% for the R, and from 78 to 86% for the GLPA, depending on DH population and environment. Similarly to the CH, within the agronomic environments the highest R was detected in the E3 (~ 98 mg per spike) and the lowest in the E2 (~ 60 mg per spike) for both populations (Table S6). The R measured in the E5 was even lower than the one of E2 (48 mg per spike, Table S6). The GLPA varied from ~ 216 to 564 mg per spike within the agronomic environments, reaching 234 mg per spike in the E5 (Table S6). The GW ranged from ~ 32 to 47 mg (E4-E3) for BP11xB2002 and from ~ 29 to 41 mg (E2-E5) for B19xB2002 including the non-agronomic environment (Table S6).

For both populations, when the spikes were longer (higher SL), they bore more total and fertile spikelets per spike (TS and FS) distributed in a laxly way (higher mm per node or higher CN) (Figures S1 and S2); this resulted in increased fertile florets per spike (FF). In both populations, the longer spikes were heavier (higher SDW and CH), which showed positive correlation with the FF but reduced efficiency to set fertile florets or grains per unit of spike growth (negative correlations SDW vs FFE and SDW vs FE in Figures S1 and S2). On the other hand, theses efficiencies to set florets and grains were positively correlated with the ability of a spikelet to bear florets (positive correlation and FFE vs FFFS), increasing the fertile florets and grains per spike (positive correlation FFE vs FF and FE vs GN) (Figures S1 and S2). Although the higher GN was positively correlated with the spike yield (YLD), it was negatively associated with the GW in both populations. Meanwhile, the GW contributed to YLD only in one population (BP11xB2002) not showing the same result in the other (B19xB2002) (Figures S1 and S2). It is interesting that the higher efficiencies to set fertile florets (FFE) and grains (FE) resulted in an increased efficiency to set yield per unit of spike growth at anthesis in both populations (r = 0.48 or 0.19 for FFE vs YLD/SDW *p* < 0.05; and r = 0.81 or 0.68 for FE vs YLD/SDW *p* < 0.0001, in B19xB2002 and BP11x B2002, respectively). These efficiencies are fundamental considering the limitation of assimilates for spike growth during the pre-anthesis period (for a thorough discussion see Pretini et al. [72]).

### QTL mapping analysis

A total of 305 QTL were identified across 5 environments and BLUE distributed on the 21 chromosomes (Table S7). Nevertheless, only 28 QTL were stable, i.e. present in at least 3 individual environments or BLUE analysis with a LOD > 2.5 considering a single population or a combination of both populations but with the contribution of the same germplasm (Baguette or B2002), and major, i.e. the R^2^ > 10% in one environment at least (Table 3). Those stable and major QTL were distributed on the 1A, 2A, 2B, 2D, 3A, 3B, 5A, 5B, 6A, 6B, 7A and 7B chromosomes (Table 3).

**Table 3:** Stable and major QTL identified for spike fertility related traits in both populations

Trait^a^	QTL	Pop^b^	Env^c^	Closest marker	Distance (cM)	IWGSC Ref Seq v1.0 (Mb)	LOD	Add^d^	R^2^
**SL**	*QSL.perg-2B*	BP11xB2002	BLUE	*wsnp_Ex_rep_c70228_69172301*	77.6	389.5	4.80	-3.2	16.4%
			E1	*RAC875_c27297_2153*	78.6	385.2	3.62	-3.8	11.9%
			E2	*RAC875_c27297_2153*	78.6	385.2	5.12	-3.1	16.6%
	*QSL.perg-5A*	B19xB2002	E5	*BS00022003_51*	41.2	444.8	2.84	-2.0	8.4%
			E2	*wsnp_Ex_c19647_28632894*	44.2	470.0	3.68	-2.4	11.6%
			E3	*Kukri_rep_c72046_78*	48.8	512.2	7.99	-4.0	24.0%
			BLUE	*Kukri_rep_c72046_78*	48.8	512.2	9.56	-3.4	27.8%
			E1	*BS00022818_51*	49.3	524.2	4.50	-3.9	14.9%
	*QSL.perg-7A*	BP11xB2002	E3	*Tdurum_contig16244_105*	36.1	68.9	6.57	-3.7	17.0%
			BLUE	*wsnp_Ku_c57198_60433631*	42.2	78.4	3.99	-2.7	12.4%
			E2	*wsnp_Ra_rep_c69620_67130107*	45.1	85.6	2.92	-2.2	8.5%
			E1	*wsnp_Ra_rep_c69620_67130107*	46.1	85.6	3.41	-4.3	14.5%
**TS**	*QTS.perg-2D*	BP11xB2002	E3	*Tdurum_contig17626_210*	60.5	571.5	5.02	-0.9	24.1%
			E2	*Tdurum_contig17626_210*	65.5	571.5	4.43	-0.8	17.0%
			BLUE	*Tdurum_contig17626_210*	68.1	571.5	8.32	-0.8	21.0%
			E1	*RAC875_c11093_174*	82.4	590.1	10.12	-1.0	24.8%
	*QTS.perg-3A*	B19xB2002	E5	*RAC875_c77648_367*	3.4	12.2	5.99	-1.0	15.7%
			BLUE	*Excalibur_c62042_175*	5.4	13.9	5.79	-0.7	17.2%
			E1	*Kukri_rep_c75764_60*	8.1	20.1	5.02	-0.8	20.6%
	*QTS.perg-7A*	BP11xB2002	BLUE	*Ku_c68368_1724*	138.1	701.6	3.82	-0.5	8.2%
			E1	*wsnp_Ku_c16022_24798741*	143.2	725.9	3.02	-0.5	5.7%
		B19xB2002	E3	*IAAV6957*	90.5	675.2	5.96	-0.6	19.8%
**CN**	*QCN.perg-2A*	BP11xB2002	E3	*BS00091763_51*	83.0	773.2	3.82	-0.13	14.0%
		B19xB2002	E1	*BobWhite_c17113_240*	81.1	751.6	3.06	-0.13	10.8%
			BLUE	*Excalibur_c35919_107*	83.7	754.5	5.07	-0.15	16.9%
			E5	*wsnp_Ex_c2137_4014287*	86.6	755.9	4.88	-0.20	15.1%
	*QCN.perg-5A*	BP11xB2002	E2	*wsnp_Ex_c24215_33462239*	57.1	526.6	9.44	-0.16	31.2%
			BLUE	*wsnp_Ex_c24215_33462239*	57.1	526.6	9.46	-0.16	26.5%
		B19xB2002	E3	*Kukri_rep_c72046_78*	48.8	512.2	5.48	-0.13	19.6%
**FF**	*QFF.perg-2B*	BP11xB2002	E3	*JD_c10643_840*	89.6	683.2	4.85	-3.75	14.8%
		B19xB2002	E3	*Tdurum_contig12879_1200*	64.2	712.6	7.85	-3.81	30.7%
			E2	*Kukri_rep_c68903_301*	65.1	730.2	7.15	-3.32	32.7%
			BLUE	*Kukri_rep_c68903_301*	65.7	730.2	8.67	-2.66	32.7%
	*QFF.perg-7B*	B19xB2002	BLUE	*Kukri_c51101_351*	61.2	630.1	5.12	1.73	15.7%
			E3	*Tdurum_contig47633_304*	65.2	659.7	3.00	2.07	10.0%
			E1	*Tdurum_contig4658_106*	71.7	680.2	4.56	3.61	17.2%
**FS**	*QFS.perg-2B*	BP11xB2002	E3	*BS00064318_51*	86.8	686.0	8.01	-0.78	20.2%
		B19xB2002	E3	*Tdurum_contig12879_1200*	64.2	712.6	3.28	-0.43	11.0%
			E2	*RAC875_c81984_707*	64.6	719.8	4.57	-0.52	16.8%
			BLUE	*RAC875_c81984_707*	64.6	719.8	4.64	-0.44	15.5%
	*QFS.perg-3A*	B19xB2002	E3	*Excalibur_c62042_175*	5.4	13.9	3.28	-0.41	11.6%
			E2	*Kukri_rep_c75764_60*	8.1	20.1	2.83	-0.36	9.8%
			E1	*BS00049032_51*	10.6	25.9	5.37	-0.78	23.1%
	*QFS.perg-5B*	BP11xB2002	E2	*RFL_Contig5461_683*	48.7	580.4	8.67	-0.88	32.1%
			BLUE	*RFL_Contig5461_683*	48.7	580.4	4.36	-0.46	10.9%
		B19xB2002	E2	*Vrn-B1: Excalibur_c5329_1335*	65.3	580.7	4.17	-0.47	14.3%
**FFTS**	*QFFTS.perg-5A*	BP11xB2002	E3	*wsnp_Ex_c24215_33462239*	57.1	526.6	3.25	0.11	10.1%
		B19xB2002	E3	*wsnp_CAP11_c1740_947838*	51.3	536.7	4.17	0.10	17.4%
			E1	*RAC875_rep_c107228_92*	70.5	567.7	3.12	0.13	14.3%
			BLUE	*RAC875_rep_c107228_92*	70.5	567.7	3.24	0.09	13.1%
	*QFFTS.perg-5B*	BP11xB2002	E2	*wsnp_Ku_c35090_44349517*	9.3	34.3	5.68	0.09	18.0%
			E1	*Kukri_rep_c71114_838*	12.9	70.3	3.93	0.14	12.6%
			BLUE	*Kukri_rep_c71114_838*	12.9	70.3	8.53	0.15	30.2%
			E3	*Ex_c68034_498*	15.0	21.5	6.06	0.16	20.8%
**FFFS**	*QFFFS.perg-7B*	B19xB2002	BLUE	*BS00063208_51*	61.7	637.6	5.69	0.09	19.2%
			E3	*Kukri_c100592_82*	62.7	648.1	4.41	0.10	16.3%
			E1	*RAC875_c60191_114*	71.7	697.1	5.06	0.15	21.8%
**R**	*QR.perg-2A*	BP11xB2002	E1	*wsnp_CAP8_c1580_908907*	19.6	33.3	8.51	8.0	27.1%
			BLUE	*wsnp_CAP8_c1580_908907*	19.6	33.3	5.91	4.2	14.5%
		B19xB2002	E5	*Kukri_c17467_2711*	0.0	4.7	2.54	3.3	5.3%
	*QR.perg-3B*	BP11xB2002	E2	*Jagger_c522_55*	66.2	730.2	5.21	4.4	16.4%
			E3	*Kukri_rep_c94476_152*	77.7	745.7	3.12	9.5	22.6%
			E4	*CAP12_c2348_133*	86.1	732.4	4.62	4.4	16.1%
			BLUE	*Excalibur_c18410_136*	89.3	752.1	7.65	5.2	21.1%
	*QR.perg-6A*	BP11xB2002	E3	*Tdurum_contig100733_89*	33.7	22.0	4.91	-8.4	19.1%
			BLUE	*RFL_Contig2954_548*	69.5	23.9	4.19	-3.0	7.4%
		B19xB2002	E1	*BobWhite_c3714_659*	1.8	8.0	2.66	-5.6	11.0%
			BLUE	*BS00083630_51*	0.0	5.6	7.02	-5.5	26.9%
**GLPA**	*QGLPA.perg-1A*	B19xB2002	BLUE	*wsnp_Ex_c4310_7770452*	144.4	464.3	10.85	-27	32.7%
			E1	*RAC875_c53185_802*	148.4	480.5	5.84	-30	21.3%
			E4	*RAC875_c53185_802*	148.4	480.5	5.70	-33	21.9%
	*QGLPA.perg-3A*	B19xB2002	BLUE	*Excalibur_c46600_919*	44.9	648.0	2.54	-11	5.9%
			E1	*Kukri_c18420_705*	51.2	663.2	3.13	-19	9.1%
			E5	*Tdurum_contig15928_135*	75.0	709.1	3.98	-24	11.0%
	*QGLPA.perg-5B*	BP11xB2002	BLUE	*RAC875_c60758_623*	64.3	597.2	5.11	18	13.2%
			E3	*BS00037023_51*	72.3	654.5	3.15	41	14.6%
		B19xB2002	E5	*Tdurum_contig13773_321*	69.4	595.7	6.51	36	24.4%
	*QGLPA.perg-7A*	BP11xB2002	BLUE	*Kukri_c64330_58*	33.3	62.9	3.02	-13	7.3%
			E3	*Tdurum_contig82510_556*	37.5	76.9	2.95	-33	10.0%
		B19xB2002	E1	*IACX17522*	34.8	57.9	3.73	-25	11.2%
**CH**	*QCH.perg-1A*	B19xB2002	BLUE	*wsnp_Ex_c4310_7770452*	144.2	464.3	9.55	-29	29.40%
			E4	*RAC875_c53185_802*	148.4	480.5	5.21	-37	20.9%
			E1	*BS00023126_51*	157.4	480.6	5.15	-36	22.0%
	*QCH.perg-2B*	BP11xB2002	E1	*wsnp_Ra_c4126_7552133*	84.0	409.3	5.98	-35	25.0%
			E3	*Kukri_rep_c91092_553*	84.8	442.3	3.83	-48	13.7%
		B19xB2002	E3	*wsnp_Ex_rep_c104478_89183627*	57.1	447.8	2.82	-35	10.0%
**GN**	*QGN.perg-5A*	B19xB2002	E2	*BS00083507_51*	42.2	461.5	4.56	1.8	14.6%
			E5	*BS00083507_51*	42.2	461.5	3.89	2.3	14.5%
			BLUE	*Ex_c19057_965*	44.7	473.6	5.00	1.8	17.5%
**GW**	*QGW.perg-5A*	BP11xB2002	E3	*Tdurum_contig67291_367*	78.1	573.8	5.59	3.0	20.1%
			BLUE	*Tdurum_contig67291_367*	78.1	573.8	3.12	1.2	8.1%
		B19xB2002	E3	*BS00032146_51*	79.8	615.2	3.36	1.5	12.8%
	*QGW.perg-6B*	B19xB2002	E4	*Kukri_rep_c117390_70*	43.4	127.6	7.13	-1.7	21.6%
			E2	*Kukri_c38398_164*	45.5	135.1	5.15	-1.6	14.0%
			BLUE	*Kukri_c38398_164*	45.5	135.1	5.73	-1.8	18.2%

^a ^*SL* spike length (mm), *TS* total spikelets per spike (n° spike^−1^), *CN* compactness of the spike (mm node^−1^), *FF* fertile florets per spike (n° spike^−1^), *FS* fertile spikelets per spike (n° spike^−1^), *FFTS* fertile florets per total spikelet (n° spikelet^−1^), *FFFS* fertile florets per fertile spikelet (n° spikelet^−1^), *R* rachis (mg spike^−1^), *GLPA* glume + lemma + palea + awns (mg spike^−1^), *CH* chaff (no-grain spike dry weight at harvest mg spike^−1^), *GN* grain number (n° spike^−1^), *GW* grain weight (mg)

^b^ Population BP11xB2002, Baguette Premium 11 × BioINTA 2002; B19xB2002, Baguette 19 × BioINTA 2002

^c^ Environment E1: Pergamino 2012, E2: Pergamino 2013, E3: Pergamino 2015, E4: Marcos Juárez 2015, E5: Pergamino 2016, BLUE

^d^ Add, additive effects: contribution of parent’s alleles to the larger values. The positive value of additive effect indicates that the Baguette allele increase the corresponding trait. The negative value of additive effect indicates that the BioINTA2002 allele increase the corresponding trait

For SL 19 QTL were detected (Table S7); however, three of them on chromosomes 2B (*QSL.perg-2B*), 5A (*QSL.perg-5A*) and 7A (*QSL.perg-7A*) were considered major and stable across environments. Both *QSL.perg-2B* and *QSL.perg-7A* were detected on BP11xB2002 while the *QSL.perg-5A* was present in B19xB2002 (Table 3). The increasing allele was always contributed by B2002 with an additive effect that ranged from 3.1 to 3.8 mm for *QSL.perg-2B*, from 2.0 to 4.0 mm for *QSL.perg-5A* and from 2.2 to 4.3 mm for *QSL.perg-7A* (Table 3). Significant epistatic interaction between *QSL.perg-2B* and *QSL.perg-7A* was detected (*P* = 0.042). The *QSL.perg-7A* allele from BP11 produced a greater reduction in SL in the presence of the allele from the same parent for *QSL.perg-2B* (Figure S3a).

For TS 24 QTL were identified (Table S7), but only three of them on chromosomes 2D (*QTS.perg-2D*), 3A (*QTS.perg-3A*) and 7A (*QTS.perg-7A*) were considered major and stable. The *QTS.perg-2D* was detected in BP11xB2002 while the *QTS.perg-3A* was observed in B19xB2002 (Table 3). The *QTS.perg-7A* was detected in two environments in BP11xB2002 (E1 and BLUE), and in one environment in B19xB2002 (E3, Table 3). The increasing allele for all the QTL was contributed by B2002 with an additive effect that ranged from 0.8 to 1.0, 0.7 to 1.0 and 0.5 to 0.6 total spikelets per spike for *QTS.perg-2D, QTS.perg-3A* and *QTS.perg-7A,* respectively (Table 3).

For CN, two QTL out of 22 (Table S7), on chromosomes 2A (*QCN.perg-2A*) and 5A (*QCN.perg-5A*) were considered major and stable across environments. The QTL were identified in both populations. The *QCN.perg-2A* was detected in one environment in BP11xB2002 (E3) and in three environments in B19xB2002 (E1, E5 and BLUE). The *QCN.perg-5A* was detected in two environments in BP11xB2002 (E2 and BLUE) and in one environment in B19xB2002 (E3, Table 3). The increasing allele of both QTL was contributed by B2002 and had an additive effect ranging from 0.13 to 0.20 mm per node (Table 3).

Despite being identified 18 QTL for FF (Table S7), only two on chromosomes 2B (*QFF.perg-2B*) and 7B (*QFF.perg-7B*) were considered major and stable (Table 3). The *QFF.perg-2B* was detected in one environment in BP11xB2002 (E3) and in three environments in B19xB2002 (E2, E3 and BLUE, Table 3), while the *QFF.perg-7B* was detected in three environments in B19xB2002 (Table 3). B2002 contributed the increasing allele for *QFF.perg-2B*, with and additive effect that ranged from 2.66 to 3.81 fertile florets per spike (Table 3). The increasing allele of *QFF.perg-7B* was contributed by B19 and had a significant additive effect that ranged from 1.7–3.6 fertile florets per spike (Table 3). No significant epistatic interaction between *QFF.perg-2B* and *QFF.perg-7B* was detected (*P* = 0.3416).

Three QTL for FS on chromosomes 2B (*QFS.perg-2B*), 3A (*QFS.perg-3A*) and 5B (*QFS.perg-5B*) were considered major and stable out of 23 identified in the QTL analysis (Table S7). The *QFS.perg-2B* was detected in one environment in BP11xB2002 (E3) and in three environments in B19xB2002 (E2, E3 and BLUE, Table 3). As regards the *QFS.perg-3A*, it was detected in three environments in B19xB2002 (E1, E2 and E3) (Table 3). Finally, the *QFS.perg-5B* was detected in two environments in BP11xB2002 (E2 and BLUE) and in one environment in B19xB2002 (E2, Table 3). The increasing allele was always contributed by B2002 with an additive effect that ranged from 0.4 to 0.9 fertile spikelets per spike (Table 3). No significant epistatic interaction between *QFS.perg-2B* and *QFS.perg-3A* was detected (*P* = 0.2133).

From the 21 QTL identified for FFTS (Table S7), two QTL on chromosomes 5A (*QFFTS.perg-5A*) and 5B (*QFFTS.perg-5B*) were considered major and stable. The *QFFTS.perg-5A* was detected in one environment in BP11xB2002 (E3) and in three environments in B19xB2002 (E1, E3 and BLUE) while the *QFFTS.perg-5B* was detected in four environments in BP11xB2002 (E1, E2, E3 and BLUE) (Table 3). For both QTL, the increasing allele was contributed by the Baguette parents with an additive effect that ranged from 0.09 to 0.13 and 0.09 to 0.16 fertile florets per total spikelet per spike for *QFFTS.perg-5A* and *QFFTS.perg-5B*, respectively (Table 3).

Only one QTL for FFFS on chromosome 7B (*QFFFS.perg-7B*) was major and stable from the 22 QTL identified (Table S7). It was detected in B19xB2002 (Table 3), in two environments (E1 and E3) and the BLUE (Table 3). The increasing allele was contributed by B19 with an additive effect that ranged from 0.09 to 0.15 fertile florets per fertile spikelet (Table 3).

Three QTL for R on chromosomes 2A (*QR.perg-2A*), 3B (*QR.perg-3B*), and 6A (*QR.perg-6A*) were major and stable; out of the 31 QTL identified for R (Table S7). The *QR.perg-3B* was only detected in BP11xB2002 while the *QR.perg-2A* and *QR.perg-6A* were identified in both populations. The *QR.perg-2A* was present in two environments in BP11xB2002 (E1, BLUE) and in one environment in B19xB2002 (E5), whereas the *QR.perg-6A* was detected in two environments for each DH population (Table 3). The increasing allele of *QR.perg-2A* and *QR.perg-3B* was contributed by the Baguette parents with an additive effect that ranged from 3.3 to 8.0 and 4.4 to 9.5 mg per spike, respectively. In contrast, the increasing allele of *QR.perg-6A* was contributed by B2002 with an additive effect varying from 3.0 to 8.4 mg per spike (Table 3).

Despite being detected 23 QTL for GLPA, (Table S7) only four of them, on chromosomes 1A (*QGLPA.perg-1A*), 3A (*QGLPA.perg-3A*), 5B (*QGPLA.perg-5B*) and 7A (*QGLPA.perg-7A*) were major and stable. The *QGLPA.perg-1A* and *QGLPA.perg-3A* were identified in BP19xB2002, while the *QGPLA.perg-5B* and *QGLPA.perg-7A* were detected in both populations. The *QGPLA.perg-5B* and *QGLPA.perg-7A* were present in two environments in BP11xB2002 and in one environment in B19xB2002 (Table 3). The increasing allele for *QGLPA.perg-1A*, *QGLPA.perg-3A* and *QGLPA.perg-7A* was contributed by B2002, with an additive effect ranging from 27 to 33, 11 to 24 and 13 to 33 mg per spike, respectively (Table 3). On the other hand, the increasing allele for *QGLPA.perg-5B* was contributed by the Baguette parents with an additive effect that ranged from 18 to 41 mg per spike (Table 3). Significant epistatic interaction between *QGLPA.perg-1A* and *QGLPA.perg-3A* was detected (*P* = 0.021). The *GLPA.perg-1A* allele from B19 produced a greater reduction in GLPA in the presence of the allele from both parents (B19 and B2002) for *GLPA.perg-3A*, but the effect was grater in the presence of the B19 (Figure S3b).

Although 25 QTL for CH were identified (Table S7), two of them, on chromosomes 1A (*QCH.perg-1A*) and 2B (*QCH.perg-2B*), were major and stable. The *QCH.perg-1A* was detected in B19xB2002 while the *QCH.perg-2B* was present in two environments in BP11xB2002 (E1 and E3) and in one in B19xB2002 (E3) (Table 3). In both cases, the increasing allele was contributed by B2002, with an additive effect varying from 29 to 37 mg per spike for *QCH.perg-1A* and from 35 to 48 mg per spike for *QCH.perg-2B* (Table 3).

For GN only one QTL on chromosome 5A (*QGN.perg-5A*) was major and stable out of the 18 QTL identified (Table S7). The QTL was detected in B19xB2002 (Table 3), in two environments (E2 and E5) and in the BLUE (Table 3). The increasing allele was contributed by B19 with an additive effect ranging from 1.8 to 2.3 grains per spike (Table 3).

From the 21 QTL detected in the analysis for GW, only two QTL on chromosomes 5A (*QGW.perg-5A*) and 6B (*QGW.perg-6B*) were major and stable. The *QGW.perg-6B* was present in B19xB2002 while the *QGW.perg-5A* was detected in two environments in BP11xB2002 (E3 and BLUE) and in one environment in B19xB2002 (Table 3). Baguette parents with an additive effect that ranged from 1.2 to 3.0 mg contributed to the increasing allele for *QGW.perg-5A* (Table 3). In contrast, for *QGW.perg-6B*, the increasing allele was contributed by B2002 ranging the additive effect from 1.6 to 1.8 mg (Table 3).

No QTL was considered major and stable for SDW and GST despite the 24 and 14 QTL respectively identified in the analysis (Table S7).

### Stable and major QTL regions for spike fertility and related traits

**Fig. 1:**
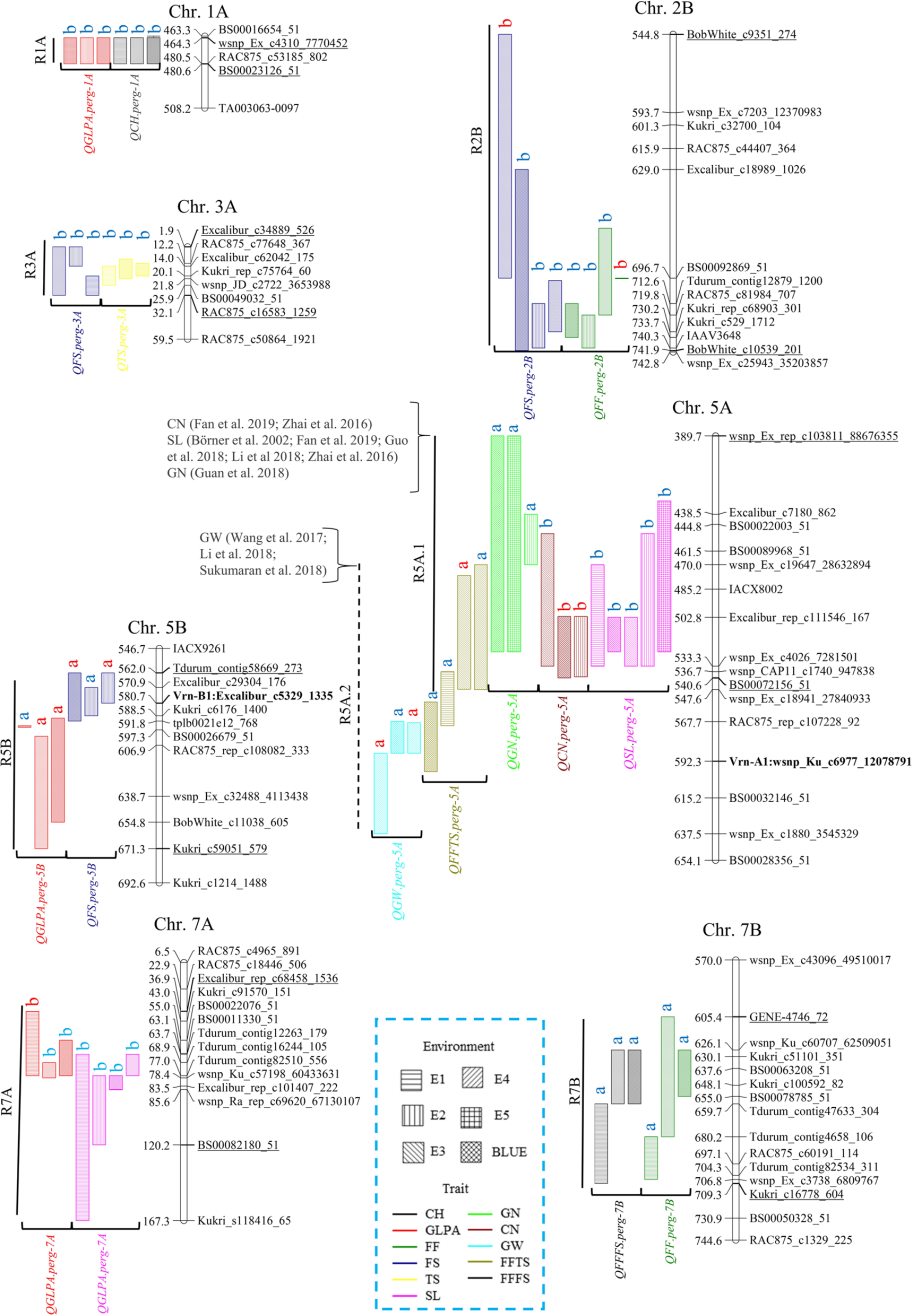
Genomic regions represented on chromosomes with markers and their reference position. *The corresponding physical distances (Mb) of the QTL regions were obtained by blasting the flanking SNP markers (± 1 LOD) of the most separated QTL in the region to the Chinese Spring RefSeq v1.0 sequence ^**^ The “a” indicates that BP11 or B19 allele increases the corresponding trait and the “b” indicates that B2002 allele increases the corresponding trait. Red letters are for the BP11xB2002 population and blue letters are for the B19xB2002 population *** Solid and dot lines indicates the markers for each hotspot **** In GN BLUE the—1 LOD SNP (Table S9) was located in a map space without markers, the closest one is 341.3 cM apart, for this reason this environment was ruled out to determine the interval of the R5A.1

Eight genomic regions distributed on seven chromosomes (R1A, R2B, R3A, R5A.1, R5A.2, R5B, R7A and R7B) were identified containing 17 of the 28 stable and major QTL detected for the different traits (Fig. 1, Tables S8 and S9). The QTL located in these regions shared a confident interval of ± 50 Mb from the SNP marker with the highest LOD value according to their physical position, indicating a potential pleiotropic effect on the corresponding traits (Fig. 1, Tables S8, S9). The increasing alleles for R1A, R2B, R3A and R7A were always contributed by B2002. The QTL peak of the R1A region was located between 464.3–480.6 Mb (± 1 LOD) and harboured *QCH.perg-1A* and *QGLPA.perg-1A*; the QTL peak of the R2B region was located between 544.8–741.9 Mb (± 1 LOD) and harboured *QFF.perg-2B* and *QFS.perg-2B*; the QTL peak of the R3A region was located between 1.9–32.1 Mb (± 1 LOD) and harboured *QTS.perg-3A* and *QFS.perg-3A*, and the QTL peak of the R7A region was located between 36.9–120.2 Mb (± 1 LOD) and harboured *QSL.perg-7A* and *QGLPA.perg-7A* (Fig. 1). On the other hand, the Baguette parents contributed the increasing alleles for the R5A.2 and R7B regions. The QTL peak of the R5A.2 region was located between 470.0–637.5 Mb (± 1 LOD) and harboured *QFFTS.perg-5A* and *QGW.perg-5A* and, the QTL peak of the R7B region was located between 605.4–709.3 Mb (± 1 LOD) and harboured *QFF.perg-7B* and *QFFFS.perg-7B* (Fig. 1). Different parents depending on the trait contributed the increasing allele for R5A.1 (Fig. 1). The QTL peak of the R5A.1 region was located between 389.7–540.6 Mb (± 1 LOD) and harboured *QSL.perg-5A* and *QCN.perg-5A* with B2002 as the increasing parent and *QGN.perg-5A* with the B19 as the increasing parent. Finally, the QTL peak of the R5B region was located between 562.0–671.3 Mb (± 1 LOD) and harboured *QFS.perg-5B* and *QGLPA.perg-5B*. In this case, the increasing alleles were contributed by B2002.

## Discussion

Most of the breeding progress in wheat yield potential has been achieved by selection of yield per se due to the lack of reliable secondary traits and molecular information available to be used in marker assisted selection (MAS) [7]. The yield potential improvement was, in most cases, consequence of increased GN [8–15], though effects of GW have been reported recently [16–18]. In the present paper we identified one stable and major QTL for GN on chromosome 5A (*QGN.perg-5A*) mapping in the same position that the one reported by Guan et al. [28]. Nevertheless, we recently reported this position as primarily controlling the fertile floret efficiency (FFE, fertile florets per g SDW) when the *QFFE.perg-5A* was identified and validated [72]. Then, as the GN is the result of the FFE and GST (both defining FE) together with the SDW [76, 77], the *QFFE.perg-5A* can be detected as a QTL associated with GN, highlighting the relevance of FFE and the QTL validated to define GN. This result exemplifies the importance of dissecting the traits into simpler and more heritable components because it enables a better search for the actual candidate gen in further research.

In relation to GW, we detected two QTL, one on chromosome 5A and other on chromosome 6B (Table 3). The first one has already been reported [24, 26, 47], but the second one, the *QGW.perg-6B* is novel. This QTL is located 157.7 Mb apart from the homeologous gene of GW2 in B genome (*GW2-B1*), associated with grain size [78], suggesting that it would not be a candidate gene to explain the phenotypic variations observed.

The GN is a complex trait itself, being the result of many numerical and physiological spike fertility related traits. In the present study, 25 major and stable QTL for spike fertility and related traits were detected (without considering the one for GN and the two of GW already mentioned in the previous paragraph). There were only two traits, SDW and GST, for which no stable and major QTL were detected. This agrees with the low narrow-sense heritability observed (see Table 2) and highlights the high impact of environment on those traits (see Table 3 in Pretini et al. [63]). Considering the three QTL detected for SL, the *QSL.perg-2B* is 13.4 Mb apart from the one already described by Cui et al. [41] (Table S1), and the *QSL.perg-5A* is located in the same region as a previously reported QTL [26, 29, 32, 35, 36] (Table 1). In contrast, no equivalent regions have been detected in previous studies for the *QSL.perg-7A*. Regarding the three QTL identified for TS, the *QTS.perg-2D* partially overlaps with one previously reported [54] (Table S1). Meanwhile, the *QTS.perg-7A* is in the same region as a previously identified QTL [27, 31, 32, 36, 38, 39, 41], (Table 1), and co-localizes with the recently described *WAPO-A1* gene (674.07 Mb) that modifies the total number of spikelet per spike [79]. Finally, the *QTS.perg-3A* detected in our work has not been previously described. In relation to the CN, the *QCN.perg-5A* is in the same region as a previous detected QTL [32, 36], but the *QCN.perg-2A* is a novel one.

Interestingly, the two QTL detected for FF are novel. The *QFF.perg-2B* is approx. 539 Mb apart from the QTL for FF detected by Guo et al. [45] discarding that it is the same region, while for *QFF.perg-7B*, no equivalent regions have been reported previously. For the FS, only the *QFS.perg-2B* is 540 Mb apart from another QTL detected previously [27, 34] (Table 1). The remaining two QTL, *QFS.perg-3A* and *QFS.perg-5B*, do not share their regions with other previous works.

For the rest of the traits analysed in this study (FFTS, FFFS, R, GLPA and CH), no previous reports are available to the best of our knowledge (Table S1). Then, we consider the QTL for FFTS (*QFFTS.perg-5A* and *QFFTS.perg-5B*), FFFS (*QFFFS.perg-7B*), R (*QR.perg-2A*, *QR.perg-3B* and *QR.perg-6A*), GLPA (*QGLPA.perg-1A*, *QGLPA.perg-3A*, *QGLPA.perg-5B* and *QGLPA.perg-7A*) and CH (*QCH.perg-1A* and *QCH.perg-2B*) are novel. No QTL was detected on chromosome 2A for FFTS or FFFS, in which the *GNI-A1* gene [80], known to increase the number of grains through higher fertile florets per spikelet, has been identified.

As many of the spike fertility traits detected in this study had similar positions, we identified eight genomic regions that share 17 major and stable QTL for the different traits (R1A, R2B, R3A, R5A.1, R5A.2, R5B, R7A and R7B). Only in two of these regions (R5A.1 and R5A.2) other QTL for the same trait have been previously described. The remaining six regions are identified for the first time as important hot spots for spike fertility traits (Fig. 1). Interestingly, the R5A.1 region, which contains QTL for SL, CN and GN, is close to the *QFFE.perg-5A* identified and validated for fertile floret efficiency in Pretini et al. [72]. The allele of B2002 parent increases the SL and CN while the allele from B19 parent increases the GN via *QFFE.perg-5A*. These results agree with the performance of the parental lines described in the present study (Table 2).

The region R5A.2, which includes *QFFTS.perg-5A* and *QGW.perg-5A,* coincides with the location of the vernalization response gene *Vrn-A1*; while the R5B region, which includes *QFS.perg-5B* and *QGLPA.perg-5B*, coincides with the location of the other vernalization response gene *Vrn-B1.* The three parental lines of the two DH populations used in the present study are spring wheats (*Vrn-A1b*/*vrn-B1* /*vrn-D1* for B19 and BP11 and *vrn-A1* /*Vrn-B1* /*vrn-D1* for B2002); and mostly insensitive to photoperiod (*Ppd-D1a* allele). This agrees with the very close anthesis dates of the lines within each population described in Pretini et al. [72], except for the summer sowing (E5) of B19xB2002 population, in which the range was higher. Furthermore, to test the effect of the two genes, we made an ANOVA using the functional markers for *Vrn-A1* and *Vrn-B1* as source of variation for time to anthesis and small differences were detected between the anthesis dates for both populations. In BP11xB2002, only five to seven days difference in time to anthesis was associated to the allelic constitution of *Vrn-A1* or *Vrn-B1*, respectively (Table S10). Similarly, for B19xB2002, three days difference in anthesis date was detected depending on the allelic constitution for both genes (Table S10). No epistatic interaction was observed between *Vrn-A1* and *Vrn-B1* in BP11xB2002, while a difference of up to 7 days to anthesis was observed depending on the allelic constitution in the B19xB2002. Based not only on those results but also on the fact that most of the QTL included in the R5A.2 and R5B regions were not expressed in the summer sowing of the environment E5 (except for *QGLPA.perg-5B* in B19xB2002), these QTL are not considered to be masking an important phenology effect. In contrast, it could be indicating that the *Vrn-A1* and *Vrn-B1* allelic variation in the population might have a pleiotropic effect on the spike traits located in those regions, with little impact on phenology in the tested conditions.

**Fig. 2:**
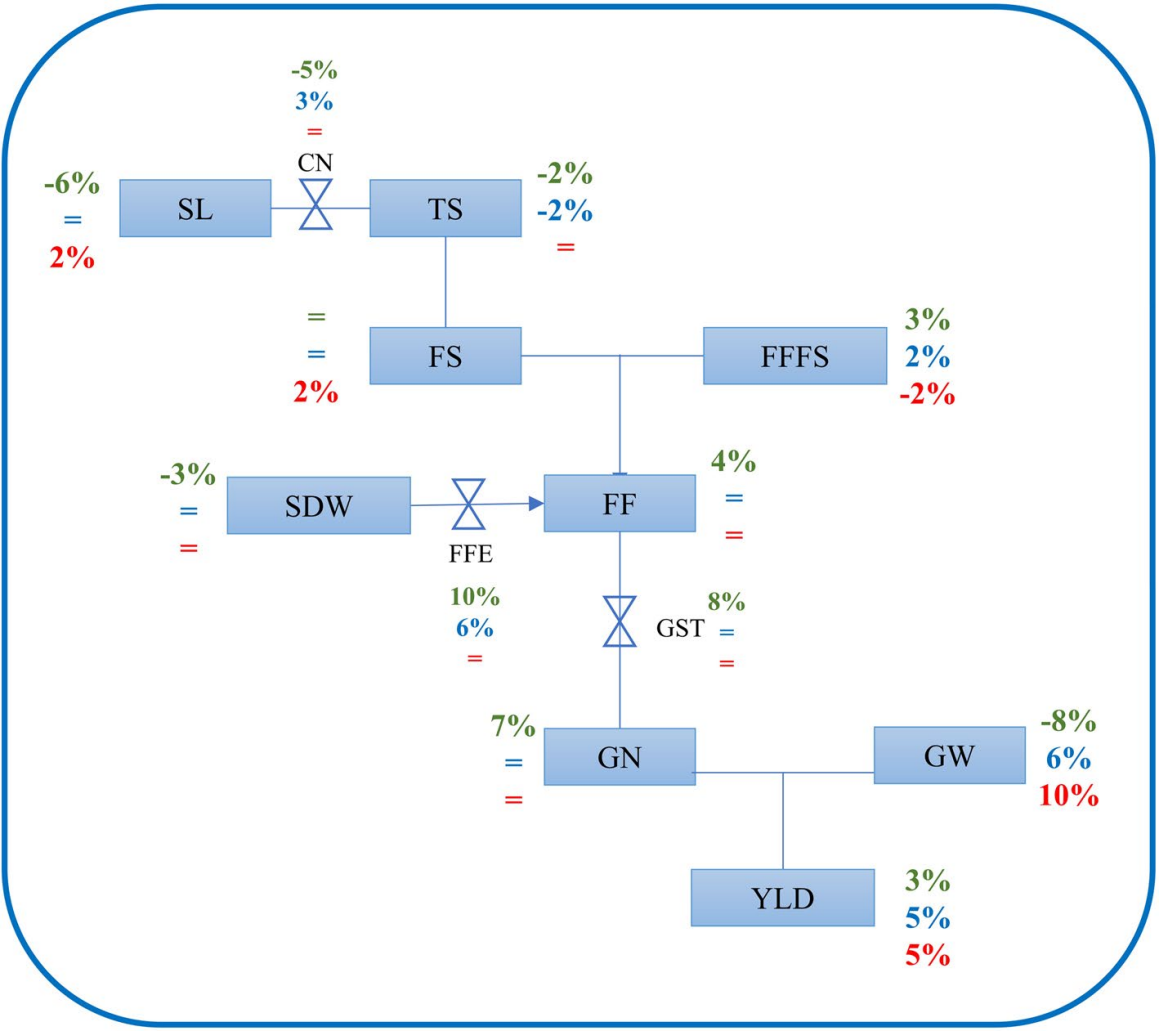
Physiological conceptual framework of measured variables showing the main and pleiotropic effects of the R5A.1 and R5A.2 regions when the allele B19 is present and *QGW.perg-6B* when the allele B2002 is present. The symbols = indicate not significant effect. The green percentage represents R5A.1 while the blue percentage represents R5A.2 and the red percentage represents *QGW.perg-6B*. SL: spike length, TS: total spikelets per spike, CN: compactness of the spike, FF: fertile florets per spike, FS: fertile spikelets per spike, FFFS: fertile florets per fertile spikelet, SDW: spike dry weight at anthesis, FFE: fertile floret efficiency, GN: grain number per spike, GW: grain weight, GST: grain set, YLD: yield

The spike fertility and related traits are correlated, positively or negatively, depending on the trait (Figures S1 and S2) [81]. In addition, a negative correlation is usually observed between GN and GW [11, 16, 82], which was also present in our data set. Then, we enquired about the possible pleiotropic effects of each of the eight regions detected over the other spike related traits, GW and final yield per spike (YLD), following the Fig. 2. For this reason, we performed an ANOVA for each of the evaluated traits using the QTL peak marker as fixed and the environments as random class variables in the model. Four regions had a significant effect on GN (R2B, R3A, R5A.1 and R5A.2), six on GW (R1A, R2B, R5A.1, R5A.2, R7A.1, R7A.2, and R7B), but only two in spike YLD (R5A.1, R5A.2). For the R5A.1 region (*QSL.perg-5A*, *QCN.perg-5A* and *QGN.perg-5A)* when the QTL from B19 is present, it results in a shorter spike (-6% SL) with similar TS (- 2%) or FS (ns), due to a reduction in the distance between spikelets (-5% CN). The FF increases 4% due to higher FFE (+ 10%), despite a reduction in the SDW (-3%) which is accompanied by a 3% increment of the FFFS. The FF increment together with the higher GST (+ 8%) results in an increment in the GN (+ 7%), which translates into higher yield (+ 3%) despite a significant reduction in GW (Fig. 2). As we previously mentioned, this region includes the *QFFE.perg-5A* identified and validated for fertile floret efficiency in Pretini et al. [72], also within the B19xB2002 population and showed similar pleiotropic effects to the R5A.1 region. The other region that resulted in a final higher YLD was the R5A.2, which contained the *QGW.perg-5A* and *QFFTS.perg-5A*. When this region from B19 is present, the SL is not affected, but the distance between spikelets is increased (+ 3% CN), reducing the TS (-2%). The FFTS and the FFFS increase 3 and 2%, respectively and the FFE is higher (+ 6%). Nevertheless, the GN is not significantly improved. The YLD improvement of R5A.2 (+ 5%), when B19 alleles are present, is consequence of the increased GW (+ 6%) (Fig. 2). As far as we know, the pleiotropic effect of these regions had not been previously reported, except for Pretini et al. [72] for the *QFFE.perg-5A,* which is within the R5A.1. We made a similar analysis of pleiotropic effect for each QTL identified (data not shown), being the *QGW.perg-6B* the only one that has a pleiotropic effect in YLD. When the B2002 alleles are present, the spikes are longer (+ 2% SL), but the TS and CN are not significantly modified. Nevertheless, higher FS were detected (+ 2%), which was counterbalanced by a reduction in the FFFS (-2%) resulting in no impact on GN. The YLD increment (+ 5%) was consequence of the increased GW (+ 10%). This is an interesting result highlighting the relevance of this QTL for the first time.

## Conclusion

From the 14 analysed traits, only two of them did not show major and stables QTL (SDW and GST). For the rest of the 12 traits, there were up to 28 significant and stable QTL and 8 hotspot regions detected. Based on the complex pleiotropic analysis preformed it is concluded that the R5A1 and R5A.2 regions together with the *QGW.perg-6B* are of high relevance to be used in MAS to improve a set of traits related with spike yield potential. All the QTL identified for the spike related traits are the first step to search for their candidate genes, which will allow their better manipulation in the future.

## Methods

### Plant materials

Two doubled haploid (DH) populations were developed from the crosses between Baguette Premium 11 × BioINTA 2002 (BP11xB2002) and Baguette 19 × BioINTA 2002 (B19xB2002). BP11xB2002 consisted of 81 lines whereas B19xB2002 consisted of 102 lines. The three parent lines are semi-dwarf hard hexaploid wheat cultivars and are adapted to central area of wheat production in Argentina (north of Buenos Aires and south of Córdoba provinces). BP11 and B19 were released by Nidera Semillas in 2004 and 2006, respectively, in Argentina, while B2002 (BPON/CCTP- F7-7792–122(87)) was developed by CIMMYT (Centro Internacional de Mejoramiento de Maíz y Trigo) and released in 2006 in Argentina by INTA. Cycles to anthesis in optimal sowing dates are similar for the three parents [59]. The GW of B2002 was higher compared to the one of BP11 and B19, whereas the GN of B19, followed by BP11, was higher than that of B2002 [68].

Generally, B2002 showed higher SDW and CH than B19 and BP11 [63, 68, 72]. Both populations were genotyped and evaluated in four (BP11xB19) or five (B19xB2002) environments (E1-E5, Table 4).

**Table 4:** Characteristics of the studied environments. Growing period, location and traits phenotyped for each DH population

Env ^a^	Growing season	Location	DH population ^b^	Traits phenotyped ^c^
E1	2012	EEA Pergamino	B19xB2002 BP11xB2002	SDW, FF, SL, TS, FS, FFTS, FFFS, CN, CH, R, GLPA
E2	2013	EEA Pergamino	B19xB2002 BP11xB2002	SDW, FF, SL, TS, FS, FFTS, FFFS, CN, CH, R, GLPA, GN, GW, GST
E3	2015	EEA Pergamino	B19xB2002 BP11xB2002	SDW, FF, SL, TS, FS, FFTS, FFFS, CN, CH, R, GLPA, GN, GW, GST
E4	2015	EEA Marcos Juárez	B19xB2002 BP11xB2002	CH, R, GLPA, GN, GW
E5	2016	EEA Pergamino	B19xB2002	SDW, FF, SL, TS, FS, FFTS, FFFS, CN, CH, R, GLPA, GN, GW, GST

^a^ Environment. All were field conditions except for E5. E1: Pergamino 2012, E2: Pergamino 2013, E3: Pergamino 2015, E4: Marcos Juárez, E5: Pergamino 2016 summer greenhouse

^b^ BP11xB2002: Baguette Premium 11 × BioINTA 2002, B19xB2002: Baguette 19 × BioINTA 2002

^c^ *SDW* spike dry weight at anthesis, *FF* fertile florets per spike, *SL* spike length, *TS* total spikelets per spike, *FS* fertile spikelets per spike, *FFTS* fertile florets per total spikelet, *FFFS* fertile florets per fertile spikelet, *CN* compactness of the spike, *CH* chaff (no-grain spike dry weight at mattradurity), *R* rachis, *GLPA* glume + lemma + palea + awns, *GN* grain number per spike, *GW* grain weight, *GST* grain set

### Experiments and phenotyping

The DH populations were grown in two experimental sites: EEA Pergamino (33^*◦*^ 51’S, 60^*◦*^ 56’W) and EEA Marcos Juárez (32^*◦*^ 43’S, 62^*◦*^ 06’W) Research Stations of INTA (Instituto Nacional de Tecnología Agropecuaria, Argentina) (Table 4). The field trails were carried out during three cropping seasons in Pergamino (E1: 2012, E2: 2013 and E3: 2015) and one cropping season in Marcos Juárez (E4: 2015) (Table 4), using a randomized complete block design (RCBD) with two replicates. Two double-row plots (1 m long and 0.21 apart, 190 plants m^−2^, E1) or five-row plots (2 m long and 0.20 apart, 330 pl m^-2^, E2-E4) were sown in optimal sowing dates. Only for B19xB2002, a fifth environment during 2016 (E5) was performed under a greenhouse during the summer season in Pergamino. After vernalization (20 days at 5^*◦*^C, 8 h light) plats were transplanted into pots during February, using a randomized complete block design (RCBD) with six replicates. Further details can be found in Pretini et al. [72].

When plants reached anthesis stage (Z6.1, [83]) five to three median spikes were selected from a larger sample (0.5 m from central row) in E1, E2 and E3, while three main stem spikes were chosen in E5. No samples were taken in E4 at anthesis. The spike length (SL, mm), the number of total spikelets per spike (TS), the number of fertile spikelet (FS), and the number of fertile florets per spike (FF) were measured following the methodology described in Pretini et al. [63]. The ratio between SL and TS was used to determine the spike compactness (CN, mm spikelet node^−1^), whereas the ratios FF/TS and FF/FS were used to estimate the number of fertile florets per total spikelet per spike (FFTS) and the number of fertile florets per fertile spikelet per spike (FFFS), respectively. After drying in an oven at 70^*◦*^C for 76 h the spike dry weight at anthesis (SDW) was estimated.

When plants reached maturity (Z9, [83]) a second spike samples were performed in a similar procedure as the one described for anthesis (including E4 environment).

Before threshing by hand, all the spikes were dried in an oven and weighted. For E1, E3, E4 and E5, the rachis (R) and the rest of the no-grain parts (glume + lemma + palea + awns, GLPA) were separated when threshing and weighted. The chaff (no-grain spike dry weight at maturity) was calculated as the sum of R + GLPA. For E2 the chaff was estimated by subtracting the weight of all the grains from the dry weight of the spike before threshing, because no chaff dissection was performed. The grain number (GN) of each spike was counted in E2, E3, E4 and E5 using an automatic counter. The grains from E1 were discarded because they were severely affected by Fusarium head blight. The grain weight (GW) was estimated as the ratio between the weight of all grains and the GN. The grain set (GST) was estimated by the ratio between GN/FF. All the phenotypic data used in this work for both populations is available in Tables S11 and S12.

### Data analyses

For each DH line, the mean value of each trait was calculated across the two replicates for E1 to E4 and the six replicates for E5. The Shapiro–Wilk test and the quantile–quantile (q-q) plot was performed to test for normal distribution. The analysis of variance (ANOVA) was performed using the Infostat/p software [84]. In addition, Best Linear Unbiased Estimator (BLUE) was estimated for each DH line including all tested environments; as random variable using R v3.3.2 and the Pearson’s correlations with the BLUE values were made to determine the relationship between all traits. The narrow-sense heritability of the traits was calculated as:

$${h}^{2} =\frac{{\sigma}_{G}^{2}}{\left({\sigma}_{G}^{2} +\frac{{\sigma}_{GE}^{2}}{E}+\frac{{\sigma}_{RES}^{2}}{ER}\right)}$$

where σ^2^_G_ is the genotypic (additive) variance, σ^2^_GE_ is the G × E interaction variance, E is the number of environments, R is the number of replications, and σ^2^_RES_ is the error variance [85].

### Linkage map construction and QTL analysis

The DH populations and the three parents were screened with the iSelect 90 K array containing 90,000 wheat SNP markers [86]. Additionally, *Vrn-A1* [73] and *Vrn-B1* [74] markers were added to the DH genetic map. The SNP markers with a high number of missing/ heterozygous data (> 20%) were discarded for the construction of the linkage map. SNPs with a 1:1 segregation distortion greater than 20% were also eliminated. Then, the dataset was reduced by merging SNPs markers with identical segregation with the Python script, merger.py^1^ [72]. Finally, the publicly available R package “Rqtl” [87] was used for the linkage map development. The physical position of SNPs associated with phenotypic traits was established by BLAST against the IWGSC Ref. Seq. v1.0 wheat genome assembly [22]. Complete linkage maps developed for both populations are available at the Tables S2 and S4.

The mean value of the trait in each environment and the BLUE values (which were treated like an additional environment) were used in the QTL mapping. The QTL analyses was performed with QTL Cartographer 2.5 software [88] through composite interval mapping (CIM) with the standard model. For the standard model we used a control marker number of 5, a window size of 10 cM and a forward and backward regression method with 500 permutations at *α* = 0.05. A LOD value of 2.5 was selected as a uniform threshold for all analyses. Detected QTL for a given trait with overlapping support intervals (< 50 Mb) were considered as equivalents. The QTL were considered “stable” if they were detected in a minimum of three environments and were defined as “major stable” if they present a R^2^ > 10% in one environment at least. For all evaluated traits in each individual DH population, we performed a factorial ANOVA using the peak marker for each major and stable QTL as class variables in the model, along with all possible two-way interactions in the case that more than one QTL was detected in order to determine significant epistatic interactions.

## Supplementary Information

**Additional file 1: Supplementary Table S1. **Major stable QTL for the analysed traits detected in 36 studies. **Supplementary Table S2. **Genetic linkage map constructed with the Baguette Premium 11 x BioINTA 2002 population. **Supplementary Table S3. **Description of the Baguette Premium 11 x BIOINTA 2002 DH population genetic map. **Supplementary Table S4. **Genetic linkage map constructed with the Baguette 19 x BioINTA 2002 population. **Supplementary Table S5. **Description of the Baguette 19 x BIOINTA 2002 DH population genetic map. **Supplementary Table S6. **Population distribution and parental means for each environment (E1 to E5) for Baguette Premium 11 x BioINTA2002 and Baguette 19 x BioINTA2002. **Supplementary Table S7. **QTL identified for spike fertility and related traits in Baguette Premium 11 x BioINTA 2002 and Baguette 19 x BioINTA 2002 DH populations. **Supplementary Table S8. **Genomic regions harbouring more than one major and stable QTL.** Supplementary Table S9. **+/- 1 and 2 LOD regions of the detected QTL. **Supplementary Table S10.** Effects of *Vrn-A1* and *Vrn-B1* on the anthesis date. **Supplementary Table S11.** Phenotypic data of the Baguette Premium 11 x BIOINTA 2002 DH population. **Supplementary Table S12. **Phenotypic data of the Baguette 19 x BIOINTA 2002 DH population.

**Additional file 2: Supplementary Figure S1. **Pearson correlations between the different attributes of the spike fertility and associated traits, based on a physiological framework for BP11xB2002 population. SL: spike length (mm), TS: total spikelets per spike (n° spike^-1^), CN: compactness of the spike (mm node^-1^), FF: fertile florets per spike (n° spike^-1^), FS: fertile spikelets per spike (n° spike^-1^), FFTS: fertile florets per total spikelet (n° spikelet^-1^), FFFS: fertile florets per fertile spikelet (n° spikelet^-1^), SDW: spike dry weight at anthesis (mg spike^-1^), R: rachis (mg spike^-1^), GLPA: glume+lemma+palea+awns (mg spike^-1^), CH: chaff (no-grain spike dry weight at maturity, mg spike^-1^), GN: grain number per spike (n° spike^-1^), GW: grain weight (mg), GST: grain set. * p < 0.05 (except for SDW vs FFE p=0,07), ** *p* < 0.01 and ***p < 0.001. **Supplementary Figure S2.** Pearson correlations between the different attributes of the spike fertility and associated traits, based on a physiological framework for B19xB2002 population. SL: spike length (mm), TS: total spikelets per spike (n° spike^-1^), CN: compactness of the spike (mm node^-1^), FF: fertile florets per spike (n° spike^-1^), FS: fertile spikelets per spike (n° spike^-1^), FFTS: fertile florets per total spikelet (n° spikelet^-1^), FFFS: fertile florets per fertile spikelet (n° spikelet^-1^), SDW: spike dry weight at anthesis (mg spike^-1^), R: rachis (mg spike^-1^), GLPA: glume+lemma+palea+awns (mg spike^-1^), CH: chaff (no-grain spike dry weight at maturity, mg spike^-1^), GN: grain number per spike (n° spike^-1^), GW: grain weight (mg), GST: grain set. * *p* < 0.05, ** *p* < 0.01 and ***p < 0.001. **Supplementary Figure S3. **Two-way interaction plots for a) SL between *QSL.perg-2B* and *QSL.perg-7A* and b) for GLPA between *QGLPA.perg-1A* and *QGLPA.perg-7A*. An asterisk indicates a significant simple effect (*P* < 0.05) of each gene in the presence of each allele of the other gene, by Fisher’s test.

## Data Availability

The datasets supporting the conclusions of this article are included within the article (Additional file 1).

## Abbreviations

B19: Baguette 19
B2002: BioINTA 2002
BP11: Baguette Premium 11
CH: Chaff (no-grain spike dry weight at harvest per spike, mg spike^−^^1^)
CN: Compactness of the spike (mm node^−^^1^)
DH: Doubled haploid
E1 to E5: Testing environments, see Table 4
FF: Fertile florets per spike (n*◦* spike^−^^1^)
FFFS: Fertile florets per fertile spikelet (n*◦* spikelet^−^^1^)
FFTS: Fertile florets per total spikelet (n*◦* spikelet^−^^1^)
FS: Fertile spikelets per spike (n*◦* spike^−^^1^)
GLPA: Glume + lemma + palea + awns (mg spike^−^^1^)
GN: Grain number per spike (n*◦* spike^−^^1^)
GST: Grain set (n*◦* grains floret^−^^1^)
GW: Grain weight (mg)
R: Rachis (mg spike^−^^1^)
SDW: Spike dry weight at anthesis (mg spike^−^^1^)
SL: Spike length (mm)
TS: Total spikelets per spike (n*◦* spike^−^^1^

## Footnotes

1
https://github.com/juancrescente/lmap
