# Supplementary material for: Mapping QTL for spike fertility and related traits in two doubled haploid wheat (Triticum aestivum L.) populations
Source: BMC Plant Biol. 2021 Jul 26;21:353. doi: 10.1186/s12870-021-03061-y (PMC8314532; doi:10.1186/s12870-021-03061-y)
Supplement: Supplementary file 2 — Additional file 2: Supplementary Figure S1. Pearson correlations between the different attributes of the spike fertility and associated traits, based on a physiological framework for BP11xB2002 population. SL: spike length (mm), TS: total spikelets per spike (n° spike-1), CN: compactness of the spike (mm node-1), FF: fertile florets per spike (n° spike-1), FS: fertile spikelets per spike (n° spike-1), FFTS: fertile florets per total spikelet (n° spikelet-1), FFFS: fertile florets per fertile spikelet (n° spikelet-1), SDW: spike dry weight at anthesis (mg spike-1), R: rachis (mg spike-1), GLPA: glume+lemma+palea+awns (mg spike-1), CH: chaff (no-grain spike dry weight at maturity, mg spike-1), GN: grain number per spike (n° spike-1), GW: grain weight (mg), GST: grain set. * p < 0.05 (except for SDW vs FFE p=0,07), ** p < 0.01 and ***p < 0.001. Supplementary Figure S2. Pearson correlations between the different attributes of the spike fertility and associated traits, based on a physiological framework for B19xB2002 population. SL: spike length (mm), TS: total spikelets per spike (n° spike-1), CN: compactness of the spike (mm node-1), FF: fertile florets per spike (n° spike-1), FS: fertile spikelets per spike (n° spike-1), FFTS: fertile florets per total spikelet (n° spikelet-1), FFFS: fertile florets per fertile spikelet (n° spikelet-1), SDW: spike dry weight at anthesis (mg spike-1), R: rachis (mg spike-1), GLPA: glume+lemma+palea+awns (mg spike-1), CH: chaff (no-grain spike dry weight at maturity, mg spike-1), GN: grain number per spike (n° spike-1), GW: grain weight (mg), GST: grain set. * p < 0.05, ** p < 0.01 and ***p < 0.001. Supplementary Figure S3. Two-way interaction plots for a) SL between QSL.perg-2B and QSL.perg-7A and b) for GLPA between QGLPA.perg-1A and QGLPA.perg-7A. An asterisk indicates a significant simple effect (P < 0.05) of each gene in the presence of each allele of the other gene, by Fisher’s test. [file 12870_2021_3061_MOESM2_ESM.pptx]

## Slide 1
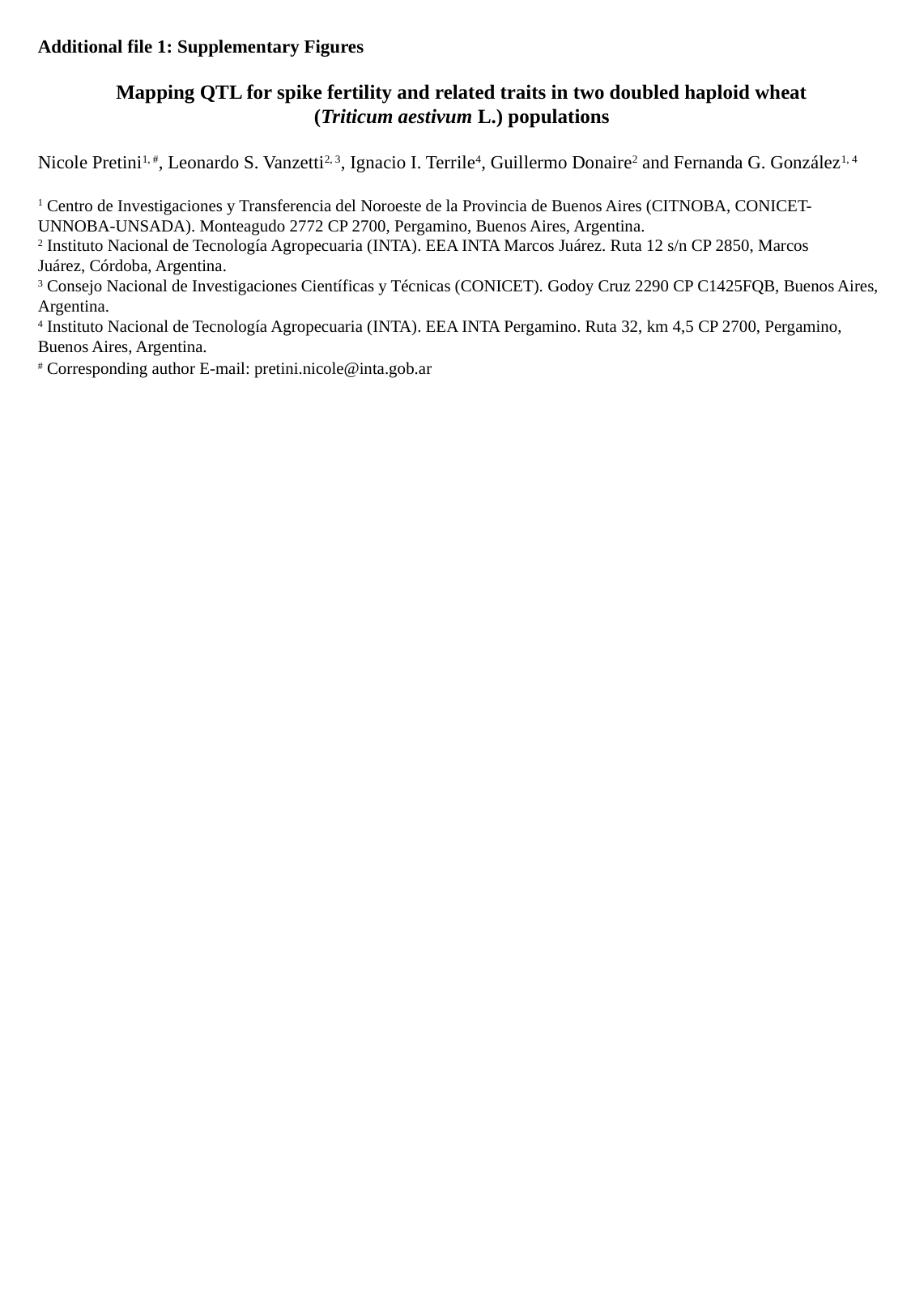

Additional file 1: Supplementary Figures
Mapping QTL for spike fertility and related traits in two doubled haploid wheat (Triticum aestivum L.) populations
Nicole Pretini1, #, Leonardo S. Vanzetti2, 3, Ignacio I. Terrile4, Guillermo Donaire2 and Fernanda G. González1, 4
1 Centro de Investigaciones y Transferencia del Noroeste de la Provincia de Buenos Aires (CITNOBA, CONICET-UNNOBA-UNSADA). Monteagudo 2772 CP 2700, Pergamino, Buenos Aires, Argentina.
2 Instituto Nacional de Tecnología Agropecuaria (INTA). EEA INTA Marcos Juárez. Ruta 12 s/n CP 2850, Marcos Juárez, Córdoba, Argentina.
3 Consejo Nacional de Investigaciones Científicas y Técnicas (CONICET). Godoy Cruz 2290 CP C1425FQB, Buenos Aires, Argentina.
4 Instituto Nacional de Tecnología Agropecuaria (INTA). EEA INTA Pergamino. Ruta 32, km 4,5 CP 2700, Pergamino, Buenos Aires, Argentina.
# Corresponding author E-mail: pretini.nicole@inta.gob.ar

## Slide 2
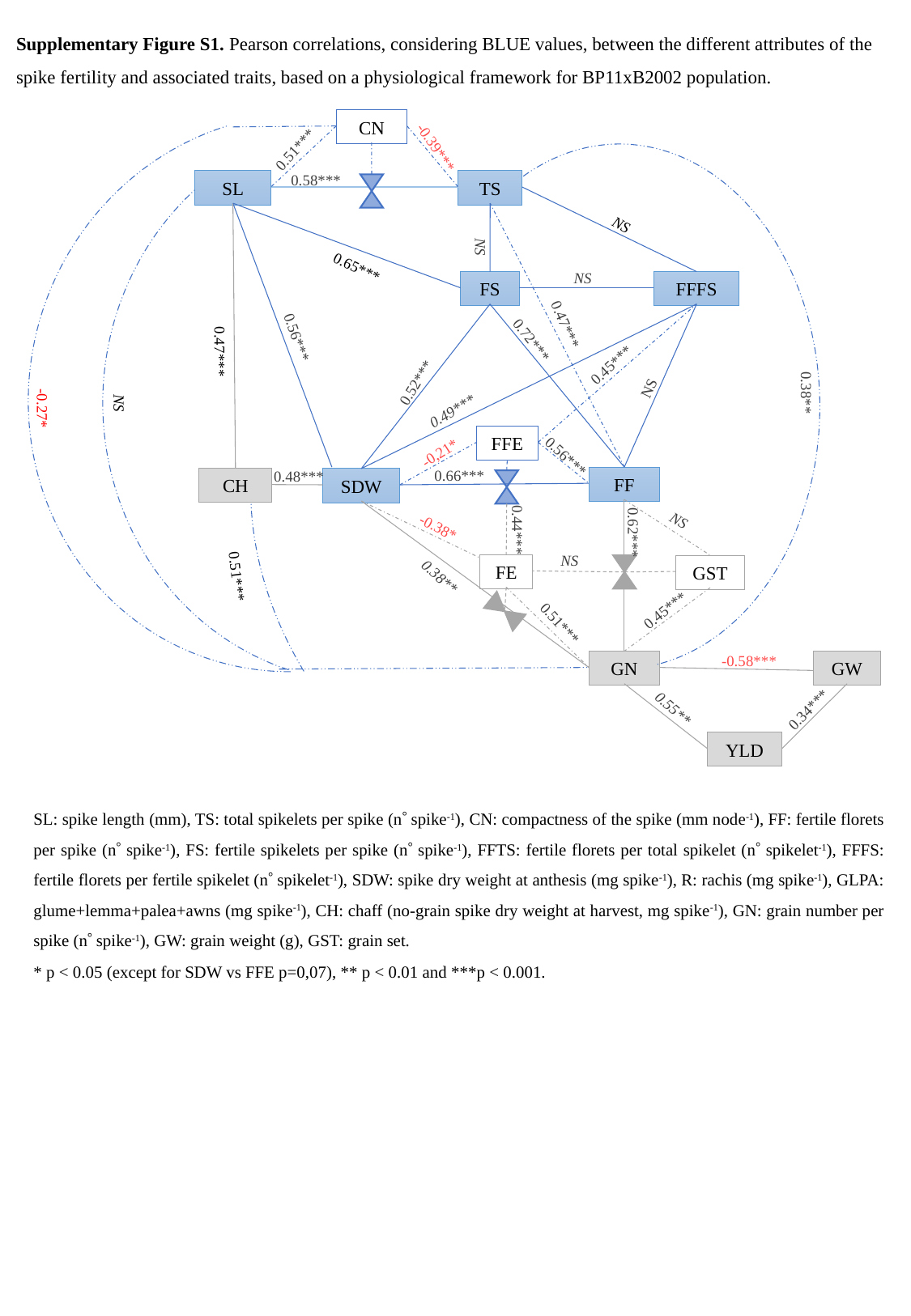

Supplementary Figure S1. Pearson correlations, considering BLUE values, between the different attributes of the spike fertility and associated traits, based on a physiological framework for BP11xB2002 population.
CN
-0.39***
0.51***
0.58***
SL
TS
NS
NS
0.65***
NS
FS
FFFS
0.47***
0.56***
0.72***
0.47***
0.45***
0.52***
NS
0.38**
NS
-0.27*
0.49***
FFE
-0,21*
0.56***
0.66***
0.48***
FF
CH
SDW
NS
-0.38*
0.44***
0.62***
NS
FE
GST
0.51***
0.38**
0.45***
0.51***
-0.58***
GN
GW
0.34***
0.55**
YLD
SL: spike length (mm), TS: total spikelets per spike (n spike-1), CN: compactness of the spike (mm node-1), FF: fertile florets per spike (n spike-1), FS: fertile spikelets per spike (n spike-1), FFTS: fertile florets per total spikelet (n spikelet-1), FFFS: fertile florets per fertile spikelet (n spikelet-1), SDW: spike dry weight at anthesis (mg spike-1), R: rachis (mg spike-1), GLPA: glume+lemma+palea+awns (mg spike-1), CH: chaff (no-grain spike dry weight at harvest, mg spike-1), GN: grain number per spike (n spike-1), GW: grain weight (g), GST: grain set.
* p < 0.05 (except for SDW vs FFE p=0,07), ** p < 0.01 and ***p < 0.001.

## Slide 3
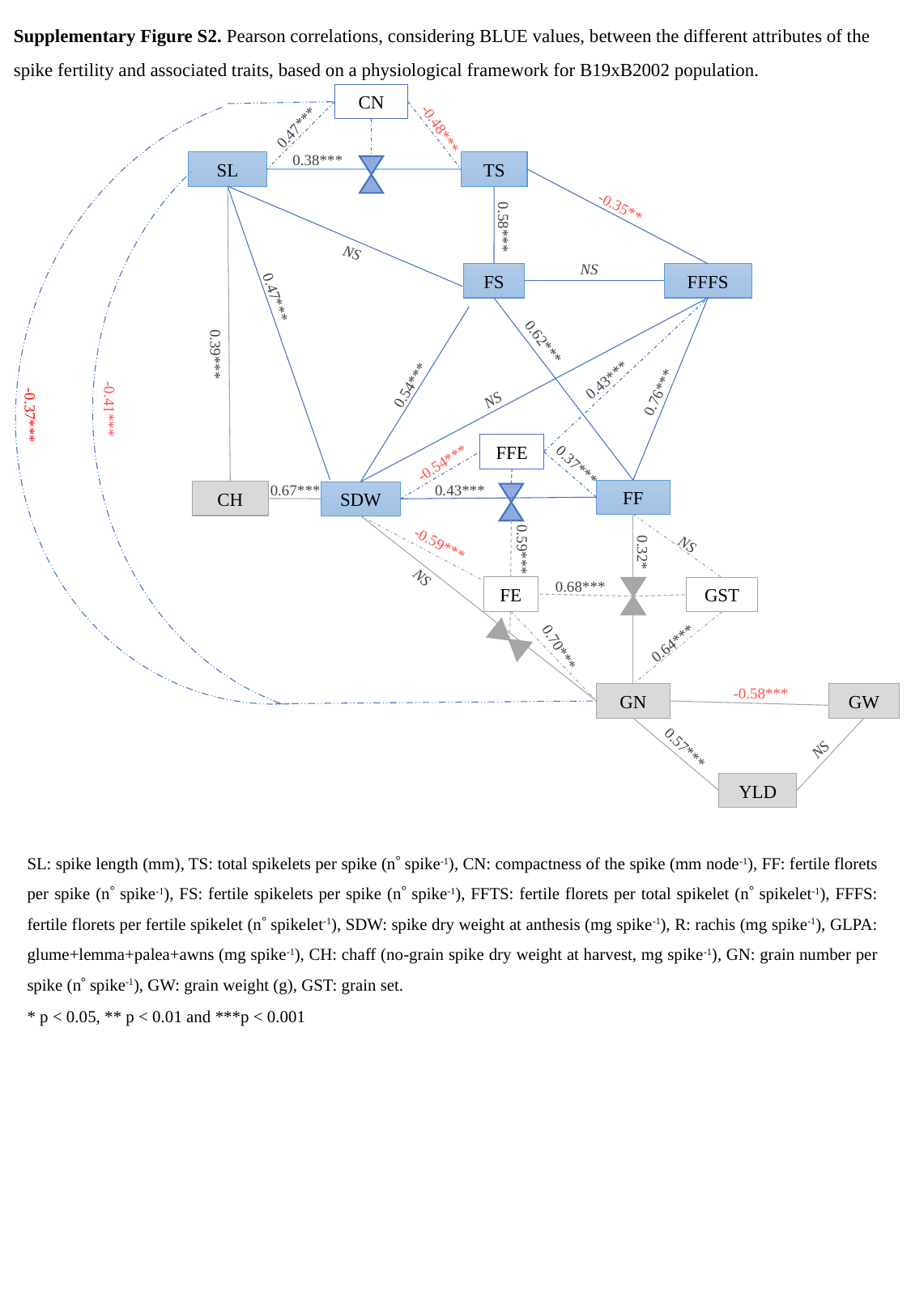

Supplementary Figure S2. Pearson correlations, considering BLUE values, between the different attributes of the spike fertility and associated traits, based on a physiological framework for B19xB2002 population.
CN
0.47***
-0.48***
0.38***
SL
TS
-0.35**
0.58***
NS
NS
FS
FFFS
0.47***
0.62***
0.39***
0.43***
0.54***
0.76***
NS
-0.41***
-0.37***
FFE
-0.54***
0.37***
0.43***
0.67***
FF
CH
SDW
-0.59***
NS
0.59***
0.32*
NS
0.68***
FE
GST
0.64***
0.70***
-0.58***
GN
GW
NS
0.57***
YLD
SL: spike length (mm), TS: total spikelets per spike (n spike-1), CN: compactness of the spike (mm node-1), FF: fertile florets per spike (n spike-1), FS: fertile spikelets per spike (n spike-1), FFTS: fertile florets per total spikelet (n spikelet-1), FFFS: fertile florets per fertile spikelet (n spikelet-1), SDW: spike dry weight at anthesis (mg spike-1), R: rachis (mg spike-1), GLPA: glume+lemma+palea+awns (mg spike-1), CH: chaff (no-grain spike dry weight at harvest, mg spike-1), GN: grain number per spike (n spike-1), GW: grain weight (g), GST: grain set.
* p < 0.05, ** p < 0.01 and ***p < 0.001

## Slide 4
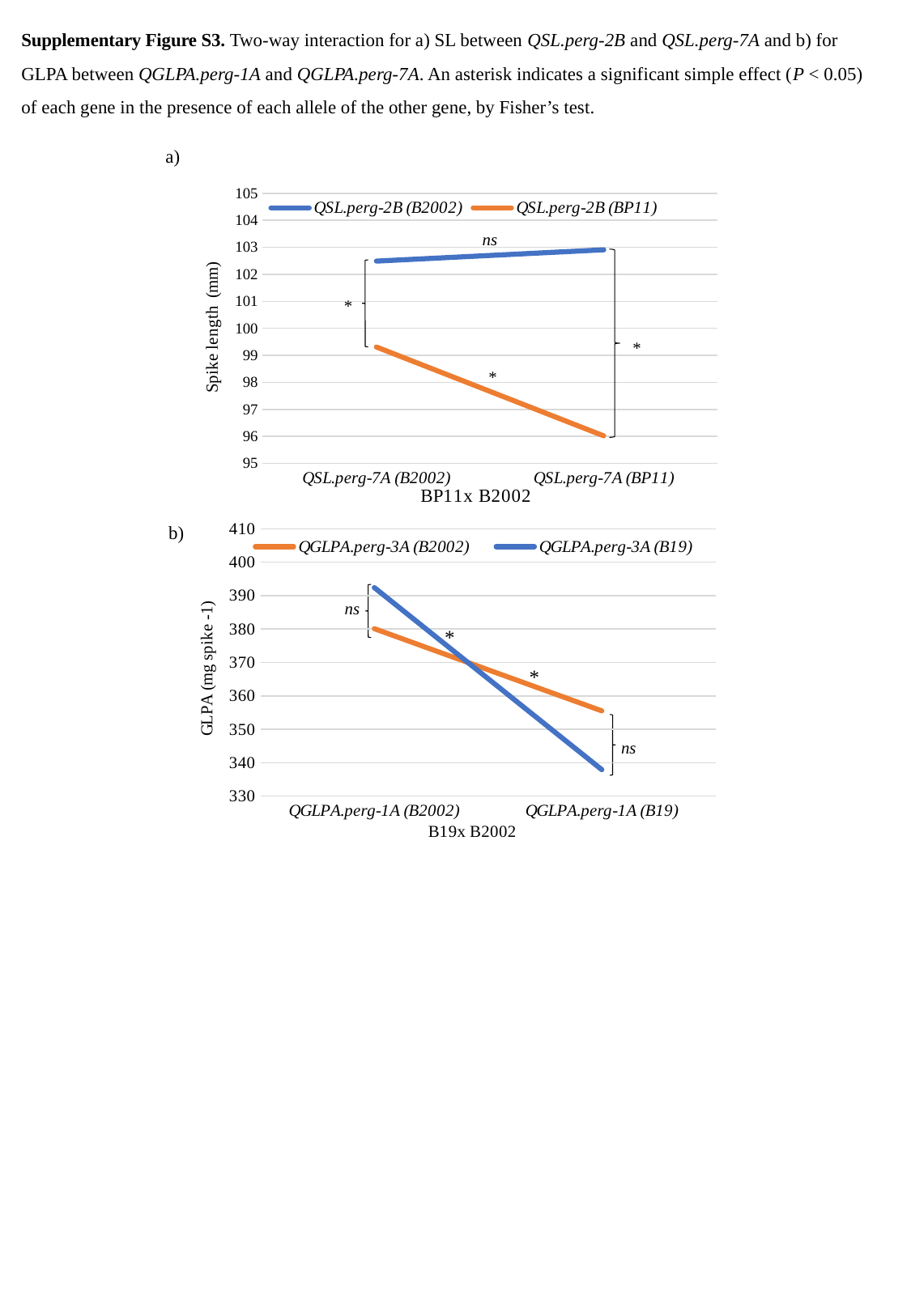

Supplementary Figure S3. Two-way interaction for a) SL between QSL.perg-2B and QSL.perg-7A and b) for GLPA between QGLPA.perg-1A and QGLPA.perg-7A. An asterisk indicates a significant simple effect (P < 0.05) of each gene in the presence of each allele of the other gene, by Fisher’s test.
a)
### Chart
| Category | QSL.perg-2B (B2002) | QSL.perg-2B (BP11) |
|---|---|---|
| QSL.perg-7A (B2002) | 102.49 | 99.31 |
| QSL.perg-7A (BP11) | 102.91 | 96.02 |ns
*
*
*
b)
### Chart
| Category | QGLPA.perg-3A (B2002) | QGLPA.perg-3A (B19) |
|---|---|---|
| QGLPA.perg-1A (B2002) | 380.1 | 392.4 |
| QGLPA.perg-1A (B19) | 355.5 | 337.9 |
ns
*
*
ns
